# Supplementary figures and images for: Modulation of Antigen Display on PapMV Nanoparticles Influences Its Immunogenicity
Source: Vaccines (Basel). 2021 Jan 8;9(1):33. doi: 10.3390/vaccines9010033 (PMC7829862; doi:10.3390/vaccines9010033)

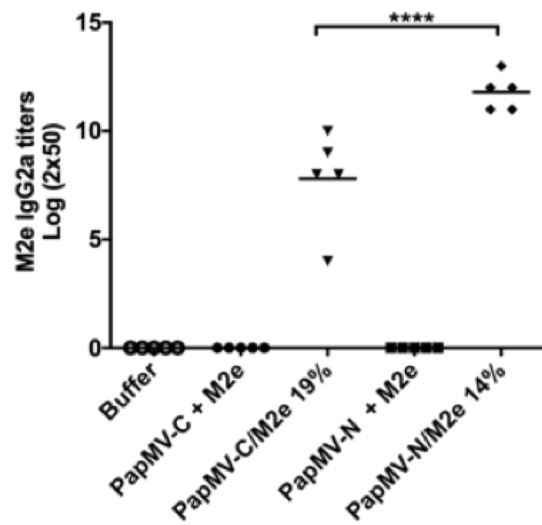

Figure S1

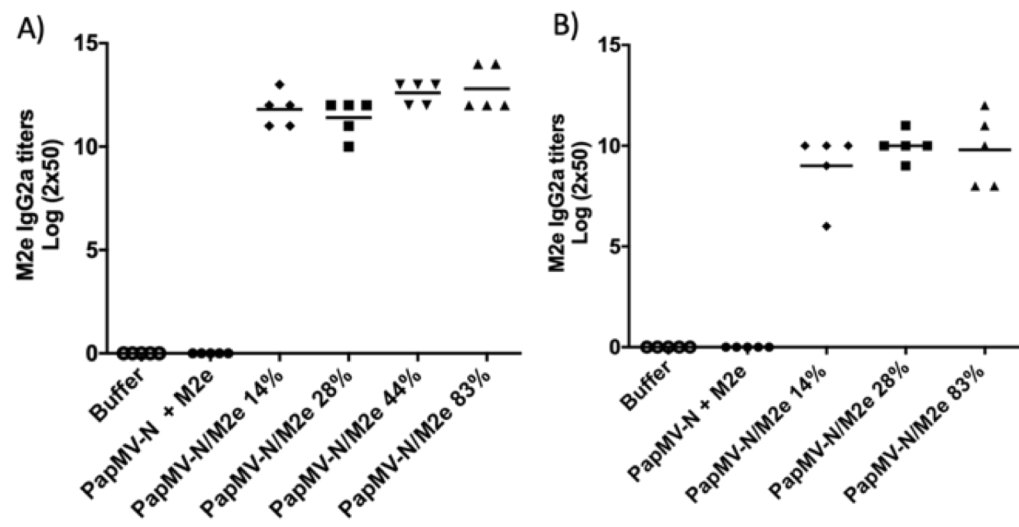

Figure S2

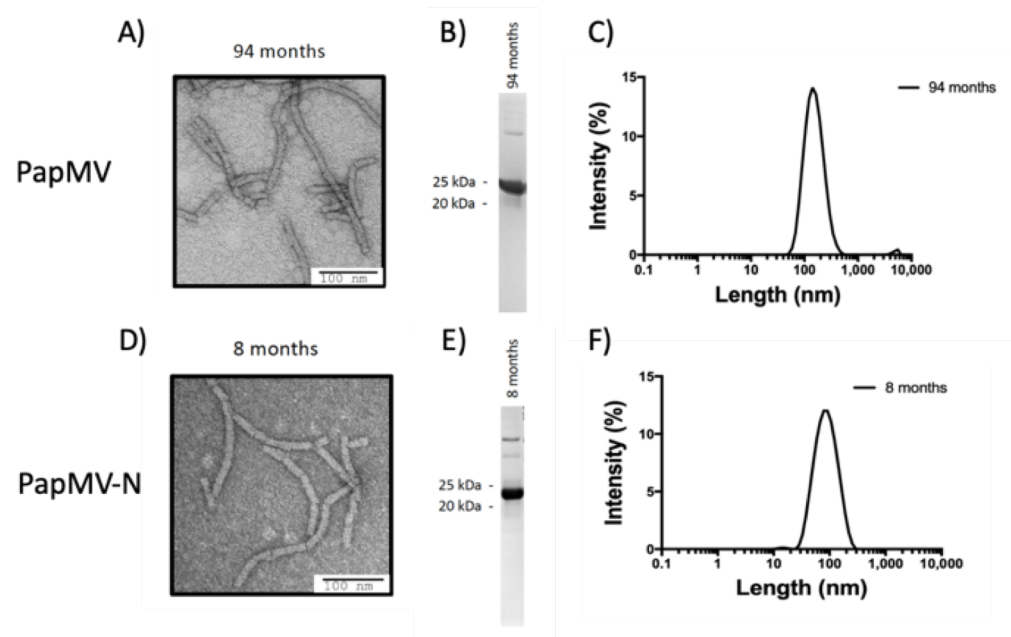

Figure S3

Supplement: Supplementary file 1 [file vaccines-09-00033-s001.pdf]
